# Quantifying the experiences of Black and Dual Heritage young people in a forensic child and adolescent mental health service

**DOI:** 10.1192/bjb.2024.74

**Published:** 2025-12

**Authors:** Michelle A. Sandiford, David Moran, Jared G. Smith, Heidi Hales

**Affiliations:** 1 North West London Forensic Child and Adolescent Mental Health Service, West London NHS Trust, London, UK; 2 Population Health Research Institute, St George's, University of London, UK; 3 West London Community Forensic Child and Adolescent Mental Health Service, West London NHS Trust, London, UK; 4 Conwy Child and Adolescent Mental Health Service, Betsi Cadwaladr University Health Board North Wales, Bangor, UK; 5 School of Medicine, Cardiff University, UK

**Keywords:** Forensic child and adolescent mental health services, child and adolescent psychiatry, forensic psychology, equality and diversity, healthcare inequalities

## Abstract

**Background:**

Young people from racialised backgrounds are overrepresented in justice services. This study explored differences in community support offered to young people from racialised groups referred to a forensic child and adolescent mental health service.

**Method:**

We compared support offered to 427 young people, according to five ethnic groups.

**Results:**

Over 20% of young people referred were Black (compared with 14% of the local population) and 15.8% were Dual White and Black Heritage (compared with 4% of the local population). Odds ratios showed that Black and Dual Heritage groups were more frequently involved with youth offending services (Black: 2.59, Dual Heritage: 2.88), gangs services (Black: 4.31, Dual Heritage: 7.13) and have a national referral mechanism (Black: 3.61, Dual Heritage: 4.01) than their White peers, but were less often in mainstream education compared with their Asian peers (Black: 0.26, Dual Heritage: 0.29). Black (odds ratio 0.35) and Dual Heritage (odds ratio 0.40) young people were less frequently diagnosed with a neurodevelopmental disorder than their White peers.

**Conclusions:**

Those from Black and Dual Heritage backgrounds were disproportionately disadvantaged.

Significant disparities are experienced across ethnicities within the youth justice system (YJS). More young people from racialised (Black, Asian and minority ethnic) groups have become involved with youth offending services (YOS), whereas youth offending overall reduced.^1^ Black and Dual Heritage young people receive more restrictive remand outcomes, less out-of-court disposal decisions and longer court sentences.^2^ They also experience disadvantages in health, social care and educational services; are less likely to be accepted for adoption;^3^ and are more likely to have unaddressed adverse childhood experiences,^4^ permanent exclusion^5^ and entry to child and adolescent mental health services (CAMHS) through compulsory routes.^6^

Steps have been taken to protect vulnerable individuals and promote safe behaviours. The national referral mechanism (NRM) is a government structure created to identify and support vulnerable individuals at risk of exploitation.^7^

Forensic CAMHS (F-CAMHS), a specialist service across England, offers consultation to professionals working with young people with complex needs and risk behaviours.^8,9^ Youth from racialised backgrounds were underrepresented in F-CAMHS in the national sample^9^ and Manchester F-CAMHS (which covers North-West England, containing both urban Manchester and rural North-West England).^10^ London, the city in which North-West London F-CAMHS is based, covers a more ethnically diverse population. According to the 2021 census estimates, youth of Black/Black British, Asian/Asian British and Dual Heritage ethnic backgrounds (aged 5–17 years) were identified in approximately 18.2, 23.3 and 11.0% of young people in London, respectively, but only 12.2, 3.7 and 4.7% in North-West England.^11^ This enabled a deeper study of disproportionality of services offered to young people from racialised groups in our area.

We aim to investigate whether the experiences of young people from Black and Dual Heritage (specifically, Black British and White parentage groups) are different to those from White and other ethnic groups.

## Method

This is a retrospective cohort study.

### Population

The population covered all young people (aged under 18 years) living in eight Boroughs of North-West London, referred to as the North-West London F-CAMHS team.

### Sample

The sample consisted of consecutive referrals since the service's inception (August 2018) through to the end of December 2022. Over this period, there were 493 referrals of young people, with 49 re-referrals. For the purposes of this study, re-referrals were considered an extension of previous input; therefore, only the first referral for each young person was included in the study.

### Ethical approval

Data collection was agreed as part of a service evaluation and quality improvement project approved by the West London NHS Trust Audit Committee. The data used were routinely collected for the service's annual report. Therefore, consent from participants was not required. Data were pseudo-anonymised at source.

### Data collection

Pseudo-anonymised data were collected by the allocated F-CAMHS team member for each referral, on demographics (age, gender, ethnicity, social circumstances), needs (emotional, mental health, social and educational), prior and current service involvement (health, social care, education and YJS), referrer agency and F-CAMHS input.

### Statistical analysis

Initially, the proportion of F-CAMHS referrals by ethnic background were compared with the corresponding population prevalence of individuals aged 5–17 years in the catchment area (data derived from the 2021 UK Census^11^), using a one proportion *z*-test.^12^ Subsequently, experiences with health, social care, education and YJS before and during the involvement with F-CAMHS were compared across five (broad) ethnic background classifications (White, Black, Asian, Arab and Dual White and Black Heritage), using *χ*^2^-tests with *post hoc* pairwise comparisons (using *χ*^2^-tests or Fisher exact tests) where significant differences were observed. Three White Travellers were excluded from analyses. Pairwise comparisons were corrected for multiple comparisons by using the false discovery rate approach, controlled at an alpha level of 5%,^13^ and odds ratios and 95% confidence intervals were calculated to describe the magnitude of effects. Sensitivity analyses within ethnic background classifications where *n* > 15 (White British versus White other, Black British African versus Black British Caribbean, Asian British Indian versus Asian British other) were carried out to establish whether results were homogeneous within (broad) classifications. Comparisons between proportions for outcomes before and during/post F-CAMHS input were measured with the McNemar mid-*P*-test for binary matched-pair data.^14^ Aside from analyses where false discovery rate was applied, the criterion for statistical significance was set at *P* < 0.05. All statistical analyses were completed with SPSS for Windows version 28.0.

## Results

A total sample of 444 young people was used in this study, 437 of which had data relating to ethnic background. A total of 343 (77.3%) participants were cis male, 95 (21.4%) were cis female, three (0.7%) were non-binary, two (0.5%) were trans male and one (0.2%) was trans female.

### Ethnic background

Over a fifth were Black and just under a sixth were Dual Heritage (most of which were Dual White and Black Heritage; Table 1). Approximately 40% were White, 14% were Asian and 9% were Arab. Relative to 2021 UK Census population estimates, there was a significant increase in odds of a White young person being referred to North-West London F-CAMHS, reflecting the 60% increase in odds of referral for a White British young person. There was a 73% increase in odds of a Black young person being referred to North-West London F-CAMHS, which was almost entirely because of overrepresentation of Black Caribbean young people, for which the associated risk of referral was particularly high. Relative to census population proportions, there was an almost three-fold increase of referral odds for White and Black Dual Heritage (referred to as Dual Heritage) youth and more than 50% increase in odds for those identifying as Arab. In contrast, the odds of an Asian young person being referred to North-West London F-CAMHS was less than half that of the corresponding census population proportion.

**Table 1:** Ethnic background of patients referred to North-West London Forensic Child and Adolescent Mental Health Service, with reference to population-based data (for those aged 5–17 years) in relevant catchment area

Ethnic background (*n* = 417)	*n*	%	Popn %	*P-*value	Odds ratio (95% CI)
**White**	**176**	**40**.**3**	**34**.**2**	**0**.**008**	**1.30** (**1.07–1.57)**
**White British**	**134**	**30**.**7**	**21**.**6**	**<0**.**001**	**1.60** (**1.31–1.97)**
White Irish	2	0.5	0.7	0.572	
White Gypsy/Traveller or Roma	3	0.7	0.5	0.498	
White Other	37	8.5	11.4	0.052	
**Black/Black British**	**93**	**21**.**3**	**13**.**6**	**<0**.**001**	**1.73** (**1.37–2.17)**
African	37	8.5	9.1	0.616	
**Caribbean**	**46**	**10**.**5**	**2**.**4**	**<0**.**001**	**4.82** (**3.55–6.54)**
Black other	10	2.3	2.1	0.745	
**Asian/Asian British**	**62**	**14**.**2**	**31**.**4**	**<0**.**001**	**0.36** (**0.28–0.47)**
**Indian**	**21**	**4**.**8**	**14**.**5**	**<0**.**001**	**0.30** (**0.19–0.46)**
Pakistani	14	3.2	5.1	0.079	
Bangladeshi	5	1.1	1.7	0.352	
Chinese	3	0.7	0.8	0.823	
**Asian other**	**19**	**4**.**3**	**9**.**3**	**0**.**001**	**0.44** (**0.28–0.70)**
**Arab/Arab British**	**37**	**8**.**5**	**5**.**4**	**0**.**006**	**1.61** (**1.15–2.26)**
**Dual Heritage**	**69**	**15**.**8**	**10**.**0**	**0**.**001**	**1.70** (**1.31–2.19)**
**White and Black/Black British**	**42**	**9**.**6**	**3**.**5**	**<0**.**001**	**2.94** (**2.14–4.05)**
White and Asian/Asian British	9	2.1	3.2	0.186	
Other	18	4.1	3.3	0.332	

Popn % refers to the ethnic background population percentage values in the catchment area served by the healthcare provider, derived from the 2021 UK Census (population values in the Office for National Statistics data release were rounded to the nearest 5, so may not reflect precise counts). Dual Heritage other included four individuals with dual White and Arab/Middle Eastern background, four individuals with dual Black (British African/Caribbean/other) and Asian background, and three individuals with dual Black British African and Black British Caribbean background. Significant differences between the observed North-West London Forensic Child and Adolescent Mental Health Service and population ethnic background percentages after correction for multiple comparisons are in bold; odds ratios and 95% confidence intervals were calculated where significant differences were observed, using frequency data from the 2021 UK Census.

### YOS involvement

**Fig. 1:**
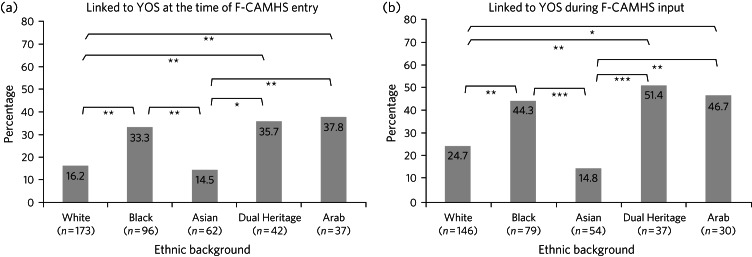
(Percentage of young people linked to youth offending services at the time of North-West London F-CAMHS entry (a) and during North-West London F-CAMHS input (b), according to ethnic background. Value labels are percentages. Asterisks indicate significant different between ethnic classifications after correction for multiple tests of association. *P < 0.05, **P < 0.01, ***P < 0.001. F-CAMHS, forensic child and adolescent mental health services; YOS, youth offending services.

There were significant differences across ethnicities regarding whether YOS was involved with a young person at the point of referral (Fig. 1a; *χ*^2^(4) = 20.53, *P* < 0.001) and during the work of North-West London F-CAMHS (Fig. 1b; *χ*^2^(4) = 25.60, *P* < 0.001). More than a third of Black, Dual Heritage and Arab youth were already involved with YOS at the point of referral, compared to one in six White young people, a more than 2.5-fold increase in odds for each (Black: odds ratio 2.59, 95% CI 1.44–4.65; Dual Heritage: odds ratio 2.88, 95% CI 1.36–6.09; Arab: odds ratio 3.15, 95% CI 1.45–6.86). Approximately half of young people with Dual Heritage and half of Arab youth were involved with YOS during North-West London F-CAMHS input, reflecting a marked increase in odds relative to White youth (Dual Heritage: odds ratio 3.23, 95% CI 1.53–6.80; Arab: odds ratio 2.67, 95% CI 1.19–6.01). Overall, YOS involvement increased from point of referral (23.8%) to discharge (31.6%; *P* < 0.001), although across ethnicities, only the differences in White (*P* < 0.001) and Black (*P* = 0.032) groups were significant.

### Gangs services involvement

**Fig. 2:**
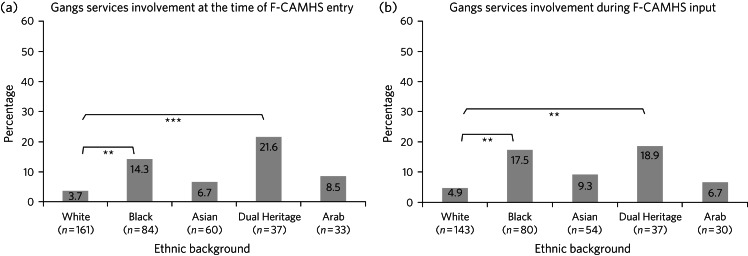
Percentage of young people with gang service involvement at the time of North-West London F-CAMHS entry (a) and during North-West London F-CAMHS input (b), according to ethnic background. Value labels are percentages. Asterisks indicate significant different between ethnic classifications after correction for multiple tests of association. **P < 0.01, ***P < 0.001. F-CAMHS, forensic child and adolescent mental health services.

There were significant associations between ethnicity and young people having an allocated gangs worker on entry to (Fig. 2a; *χ*^2^(4) = 16.97, *P* = 0.002) and discharge from North-West London F-CAMHS input (Fig. 2b; *χ*^2^(4) = 12.61, *P* = 0.013). In both cases, this reflected the greater propensity for Black young people (entry to North-West London F-CAMHS: odds ratio 4.31, 95% CI 1.55–11.93; discharge from North-West London F-CAMHS: odds ratio 4.12, 95% CI 1.59–10.70) and Dual Heritage young people (entry to North-West London F-CAMHS: odds ratio 7.13, 95% CI 2.30–22.07; discharge from North-West London F-CAMHS: odds ratio 4.53, 95% CI 1.48–13.89) to have a gangs worker relative to White youth. For White, Asian and Arab young people, gangs worker involvement was rare (<10%). Overall, gangs worker allocation did not increase significantly from point of referral (7.1%) to discharge (9.4%; *P* = 0.063).

### NRM

NRM referrals were infrequently in place at the time of entry to North-West London F-CAMHS (Table 2), although there was a significant effect of ethnicity, reflecting numerically higher rates in Black and Dual Heritage young people than other groups, which, after correction, showed no significant pairwise differences. More young people had an NRM in place on discharge from North-West London F-CAMHS (14.0%) than before referral (4.5%; *P* < 0.001). Differences across ethnic groups were heightened at discharge; compared with White young people, NRM referrals were more frequently in place for Black young people (odds ratio 3.61, 95% CI 1.63–8.00), Dual Heritage young people (odds ratio 4.01, 95% CI 1.52–10.52) and Arab young people (odds ratio 3.48, 95% CI 1.23–9.79).

**Table 2:** Social service involvement, educational status, child and adolescent mental health service/community mental health team involvement, and diagnostic and assessment status, according to ethnic background

	White (*n* = 173)	Black (*n* = 96)	Asian (*n* = 62)	Dual Heritage (*n* = 42)	Arab (*n* = 37)
	*n* (%)	*n* (%)	*n* (%)	*n* (%)	*n* (%)	*χ*^2^	*P-*value
National referral mechanism							
**On entry to North-West London F-CAMHS**	4 (2.6)	7 (8.0)	1 (1.7)	5 (13.2)	1 (3.0)	**10**.**67**	**0**.**031**
**During North-West London F-CAMHS input**	**11** (**7.2)**^b,d,e^	**19** (**21.8)**^a^	**6** (**10.3)**	**9** (**23.7)**^a^	**7** (**21.2)**^a^	**15**.**28**	**0**.**004**
Social services involvement							
On entry to North-West London F-CAMHS	130 (76.0)	78 (81.3)	45 (72.6)	38 (90.5)	30 (81.1)	6.08	0.193
**During North-West London F-CAMHS input**	**107** (**75.4)**^d^	**61** (**76.3)**	**37** (**68.5)**^d^	**35 (94.6)**^a,c^	**26** (**86.7)**	**10**.**61**	**0**.**031**
Statutory social care							
On entry to North-West London F-CAMHS	47 (27.2)	41 (42.7)	18 (29.0)	18 (42.9)	11 (29.7)	9.23	0.056
During North-West London F-CAMHS input	47 (27.2)	38 (39.6)	17 (27.4)	18 (42.9)	12 (32.4)	7.24	0.124
**In mainstream school**	**71** (**42.3)**^c^	**30** (**31.9)**^c^	**39 (63.9)**^a,b,d^	**14** (**34.1)**^c^	**16** (**43.2)**	**16**.**97**	**0**.**002**
EHCP in place							
**On entry to North-West London F-CAMHS**	**68** (**39.8)**^e^	**25** (**26.0)**	**15** (**24.2)**	**13** (**31.0)**	**5** (**13.5)**^a^	**13**.**91**	**0**.**008**
**During North-West London F-CAMHS input**	**69** (**43.1)**^e^	**28** (**30.8)**	**17** (**30.4)**	**15** (**38.5)**	**6** (**17.6)**^a^	**10**.**48**	**0**.**033**
**NEET**	**34** (**20.2)**	**31** (**33.0)**^c^	**7** (**11.5)**^b^	**6** (**14.6)**	**8** (**21.6)**	**12**.**30**	**0**.**015**
CAMHS/CMHT involvement							
**On entry to North-West London F-CAMHS**	**112 (68.7)**^b,d,e^	**48** (**51.6)**^a^	36 (61.0)	**17** (**41.5)**^a^	**16** (**44.4)**^a^	**16**.**79**	**0**.**002**
**On discharge from North-West London F-CAMHS**	**106** (**68.8)**^e^	51 (58.0)	33 (60.0)	22 (57.9)	**14** (**41.2)**^a^	**10**.**12**	**0**.**038**
Presence of diagnosis							
**On entry to North-West London F-CAMHS**	**125** (**72.3)**^e^	**58** (**60.4)**	**34** (**55.7)**	**25** (**59.5)**	**18** (**48.6)**^a^	**11**.**52**	**0**.**021**
**On discharge from North-West London F-CAMHS**	**113 (75.3)**^b,e^	**51** (**59.3)**^a^	**33** (**60.0)**	**25** (**65.8)**	**13** (**40.6)**^a^	**17**.**26**	**0**.**002**
Diagnosis type							
Conduct disorder							
**On entry to North-West London F-CAMHS**	**15** (**8.7)**	**19** (**19.8)**	**4** (**6.5)**	**8** (**19.0)**	**3** (**8.1)**	**11**.**69**	**0**.**020**
On discharge from North-West London F-CAMHS	17 (11.3)	17 (19.8)	6 (10.9)	7 (18.4)	3 (9.4)	4.92	0.295
Neurodevelopmental disorder							
**On entry to North-West London F-CAMHS**	**91 (52.6)**^b,c,d,e^	**27** (**28.1)**^a^	**19** (**31.1)**^a^	**13** (**31.0)**^a^	**8** (**21.6)**^a^	**25**.**96**	**<0**.**001**
**On discharge from North-West London F-CAMHS**	**74 (49.3)**^b,e^	**22** (**25.6)**^a^	**20** (**36.4)**	**14** (**36.8)**	**5** (**15.6)**^a^	**20**.**77**	**<0**.**001**
F-CAMHS assessment recommendation							
Attention-deficit hyperactivity disorder	17 (11.3)	17 (19.8)	4 (7.3)	5 (13.2)	3 (9.4)	5.90	0.207
Autism spectrum condition	33 (22.0)	18 (20.9)	13 (23.6)	13 (34.2)	4 (12.5)	5.02	0.286

Sample sizes differed across variables because of missing or unavailable data; reported *n* values are the maximum for any given variable. *P-*values were calculated from *χ*^2^-tests considering all ethnic background groups (significant associations are in bold ); *post hoc* pairwise comparisons were administered for those measures where an overall (significant) association was observed – significant differences after correction for multiple tests of association are indicated by superscript letters. F-CAMHS, forensic child and adolescent mental health services; EHCP, education and health care plan; NEET, not in education, employment or training; CMHT, community mental health team.

a. Significant difference from White.

b. Significant difference from Black.

c. Significant difference from Asian.

d. Significant difference from Dual Heritage.

e. Significant difference from Arab.

### Social services involvement

Social service involvement was common in young people both at entry to (76.2%) and discharge from North-West London F-CAMHS (76.5%; *P* = 0.885). Involvement significantly differed across ethnic groups in the latter period, when young people of Dual Heritage showed increased rates compared with White (odds ratio 5.72, 95% CI 1.31–25.03) and Asian youth (odds ratio 8.04, 95% CI 1.73–37.37). The proportion of young people under statutory social care remained constant from point of entry to (33.3%) and discharge from North-West London F-CAMHS input (32.4%; *P* = 0.583). Approximately 40% of Black and Dual Heritage young people were under statutory social care compared with 27–33% of White, Asian and Arab youth, although differences across ethnic groups were not significant.

### Education

There was a highly significant association between ethnicity and whether a young person was in mainstream school during North-West London F-CAMHS involvement (Table 2). *Post hoc* pairwise comparisons indicated that, compared with Asian young people, White (odds ratio 0.41, 95% CI 0.23–0.76), Black (odds ratio 0.26, 95% CI 0.13–0.52) and Dual Heritage (odds ratio 0.29, 95% CI 0.13–0.67) young people were significantly less often in mainstream school. Conversely, Asian young people were the least likely to be not in education, employment or training (NEET), although proportions were largely comparable with White and Dual Heritage youth. However, there was a more than three-fold associated risk of NEET status in Black young people relative to Asian young people (odds ratio 3.80, 95% CI 1.55–9.31). There was significant variation in whether young people had an education healthcare plan, according to ethnic background. The proportion of Arab young people with an education healthcare plan both on entry to and discharge from North-West London F-CAMHS was particularly low, with a decrease in odds of almost 80% compared with White young people (on entry to North-West London F-CAMHS: odds ratio 0.24, 95% CI 0.09–0.64; on discharge from North-West London F-CAMHS: odds ratio 0.28, 95% CI 0.11–0.72).

### Mental health

CAMHS and/or community mental health team involvement increased from the time of North-West London F-CAMHS entry (56.0%) to discharge (60.2%; *P* = 0.047), with lower rates in Black (odds ratio 0.49, 95% CI 0.29–0.82), Dual Heritage (odds ratio 0.32, 95% CI 0.16–0.65) and Arab young people (odds ratio 0.36, 95% CI 0.17–0.76) relative to White young people for the former, and in Arab young people compared with White young people for the latter (odds ratio 0.32, 95% CI 0.15–0.68; Table 2). At referral to North-West London F-CAMHS, presence of mental health diagnoses significantly differed across ethnic background groups (Table 2). Less than half of Arab young people had a diagnosis compared with almost three-quarters of White young people, an almost three-fold decrease in odds (odds ratio 0.36, 95% CI 0.18–0.75). Overall, diagnosis rates increased significantly from point of North-West London F-CAMHS entry (62.1%) to discharge (65.2%; *P* = 0.007). Differences in diagnosis rates across ethnic background groups were more obvious at discharge from North-West London F-CAMHS; both Black (odds ratio 0.48, 95% CI 0.27–0.84) and Arab (odds ratio 0.22, 95% CI 0.10–0.50) young people were significantly less likely to have a mental health diagnosis than White young people.

Neurodevelopmental disorder (NDD) diagnosis rates differed markedly according to ethnic background (Table 2). More than half of White young people had a NDD diagnosis at North-West London F-CAMHS referral, in contrast to only between a fifth and a third of young people from other ethnic backgrounds. Relative to White young people, the odds of a NDD diagnosis at North-West London F-CAMHS referral were significantly decreased for Black (odds ratio 0.35, 95% CI 0.21–0.60), Asian (odds ratio 0.40, 95% CI 0.22–0.74), Dual Heritage (odds ratio 0.41, 95% CI 0.20–0.84) and Arab (odds ratio 0.25, 95% CI 0.11–0.57) young people. Similarly, at discharge, the associated probability of NDD diagnosis was significantly reduced in Black (odds ratio 0.35, 95% CI 0.20–0.63) and Arab (odds ratio 0.19, 95% CI 0.07–0.52) young people (compared with White young people). Conversely, conduct disorder was more frequently diagnosed in Black and Dual Heritage young people than other ethnic background groups, although overall differences were only significant at time of entry to North-West London F-CAMHS and no pairwise comparisons were significant after correction for multiple tests of association. There were no significant associations between ethnicity and whether North-West London F-CAMHS recommended an attention-deficit hyperactivity disorder assessment or an autism spectrum condition assessment during their work with young people.

### Sensitivity analyses

Experiences with health, social care, education and YOS did not differ between White British and White other groups (for all comparisons, *P* > 0.101), or between Black British African and Black British Caribbean groups (for all comparisons, *P* > 0.095). NEET status was significantly more frequent in Asian British other (33.3%) than Asian British Indian (0.0%) young people (*P* = 0.006), but no other pairwise comparison yielded significant differences (for all, *P* > 0.196).

## Discussion

Corresponding to the observations made by Lammy^1^ concerning ethnic disproportionality in the YJS, we found that Black and Dual Heritage young people were overrepresented in referrals to North-West London F-CAMHS compared with the local population. A similar trend was found for Arab and White young people. In contrast, there was a marked underrepresentation of young people from Asian backgrounds in those referred. The greatest disproportionality of referral was seen for Black Caribbean young people (with a four-fold increase in odds for referral) and those of Dual Heritage (with an almost three-fold increase in odds for referral). Furthermore, analyses of service involvement highlighted that experiences of Black and Dual Heritage young people in education, health and YJS differed from those of young people from other ethnic groups; Black and Dual Heritage young people appeared to experience greater disadvantage. Dual Heritage youth had more positive support within the youth justice, social care, education and health system compared with their Black counterparts.

Notably, both nationally^9^ and for the F-CAMHS service covering Manchester and North-West England,^10^ the proportions of referrals from racialised backgrounds were much lower compared with North-West London F-CAMHS (White young people comprised three-quarters of their referrals compared with under half of ours). It is not clear whether this is wholly because of different baseline rates of young people from racialised backgrounds in the general population (as highlighted in the introduction) or whether this reflects differences in the care and support of racialised young people across England, dissimilar referring practices to different F-CAMHS services and/or a broader impact of sociodemographic status. We were not able to measure (or distinguish between) these in the present evaluation. This area of enquiry needs further study across all areas of England.

F-CAMHS aims to reduce risk of young people's involvement in the YJS, although currently, F-CAMHS involvement appears to arrive too late for Black, Dual Heritage and Arab groups compared with White (and Asian) groups. In this study, Black, Dual Heritage and Arab youth were more often already in YOS or gangs services (with allocated gangs worker) upon entry to F-CAMHS, compared with their White and Asian counterparts. This trend might reflect disproportionality in sentencing for racialised groups, particularly Black and Dual Heritage groups. It is plausible that the relatively lower level of Asian young people that are NEET compared with Black youth might mean that they are less likely to be exploited by criminals in the community and inducted into gangs, explaining why youths from these groups were less likely to be referred to gangs services than Black youths.

Dual Heritage young people appeared to have the highest rate of NRM recommendations from professionals, which can protect them from criminal exploitation. Factors contributing to this trend are unclear and require further exploration. Our findings suggest that White youth appear to be criminalised less, but also protected less by structures such as NRM when they first have contact with F-CAMHS. F-CAMHS input appears to promote NRMs across groups, suggesting an unmet need. Considering the greater tendency to be given a diagnosis of ‘vulnerability’ by CAMHS (for example, neurodevelopmental diagnoses), professionals working with White youth need to ensure that the contextual safeguarding risks of this group of young people are fully mitigated.

During F-CAMHS involvement, Dual Heritage youth were overrepresented in terms of involvement with social services compared with White and Asian groups. This corresponds to trends within the 2011 Census data.^15^ However, in contrast to the census data, the prevalence of Black young people involved with social care did not stand out compared with their peers from other ethnic groups. Although overrepresented during F-CAMHS work, Dual Heritage groups were not significantly more involved with social care at the time of their referral to F-CAMHS, suggesting an identified missed need.

The structure and pro-social routine provided by school and college is protective for young people's behaviour and emotional well-being. Black, Dual Heritage and White youth appeared to have similar rates of presence in mainstream schools. Proportionally, Asian young people have greater presence in mainstream school compared with White, Black and Dual Heritage young people. Black young people were more likely to be NEET, removing a protective factor around them.

Black and Dual Heritage youth appeared to be disadvantaged regarding their access to CAMHS compared with White youth. They were also more likely to be given a diagnosis of conduct disorder (one describing challenging behaviour with no organic basis) compared with White youth, who were more likely to receive a neurodevelopmental diagnosis (one describing difficulties and vulnerabilities). This lower diagnosis of NDD in racialised groups is also found in the Office for National Statistics survey of young people in the UK.^16,17^ White youth referred to F-CAMHS, therefore, might be protected more by CAMHS diagnoses, which are deficit and vulnerability focused, whereas there was a trend (albeit not reaching significance after correction for multiple tests) that diagnoses predominately applied to Black and Dual Heritage youth have a focus on undesirable conduct or antisocial behaviour. This difference might provide White youth with a health-based pathway to intervention and an understanding of their behaviour that is separate to the YJS. However, of the racialised groups, Dual Heritage young people appear to have the most access to this protective neurodevelopmental, health-based pathway. Furthermore, although the differences in ethnicity seen in those diagnosed with conduct disorder were reduced during involvement with F-CAMHS, suggesting that either that Black young people had a change in diagnosis away from conduct disorder or White young people had an increase in diagnosis of conduct disorder, referral for neurodevelopmental assessments was similar irrespective of ethnicity.

F-CAMHS involvement is associated with increased protection of Black, Dual Heritage and White young people, albeit via different pathways. Racialised young people are protected by government frameworks (NRM and social care), and White young people are protected by mental health diagnoses. F-CAMHS involvement was able to reframe the difficulties of Black young people into a more holistic formulation, to think about why they might present with challenging/risk behaviours.

In North-West London F-CAMHS at least, Dual Heritage young people have unique experiences compared with Black and White groups, and as such, there were benefits to keeping them as separate ethnic groups during the analyses. Dual Heritage young people are not disadvantaged when compared with White groups in terms of their access to neurodevelopmental diagnoses; furthermore, they were more likely to have a diagnosis than other racialised groups by the end of North-West London F-CAMHS input. Additionally, they had the highest rates of NRM referrals and were less likely to be NEET. It is possible that the relative advantages seen for the Dual Heritage group in CAMHS, compared with Black groups, is a product of ‘cultural capital’: through the White parent (often native to the UK) being more aware of the infrastructure of public services in the UK, having a shared language to that used in mainstream services and having had greater access to educational resources in relation to developmental disorders or mental illness. They might also implicitly be treated more favourably by services because of structural racism and unconscious bias within healthcare systems. Such hypotheses will require further exploration, as will the observed frequent advantages of Asian groups compared with other racialised groups, and the disadvantages often observed for Arab young people within public services.

### Strengths and limitations of this research

This is the first paper to focus exclusively on the experience of Black and Dual Heritage young people who were referred to F-CAMHS.

However, although unique in its ethnicity focus on F-CAMHS referrals, this study's sample size and geographical location is limited to North-West London, which may reduce the generalisability of the findings. Larger sample sizes within a broader geographic area or national studies in future will enable broader examination of the experiences of ethnicities. Further, some of the cases sampled were still open and active with F-CAMHS, so by the time of their discharge from F-CAMHS, their experiences might have been different. However, the majority of cases sampled had already been discharged from F-CAMHS, and excluding open cases from the sample would have reduced the sample size further, increasing the risk of a type 2 error. Finally, as this study is a retrospective case note investigation, it is not able to provide insights into the subjective experiences of young people from racialised groups who are referred to F-CAMHS. A future qualitative study could address this.

### Future research

Future research with larger samples will enable trends to be explored further, especially those relating to young people from Dual Heritage and Arab backgrounds. Qualitative research is needed, to explore whether behaviours of young people are viewed differently and explored differently by professionals in CAMHS according to their ethnicity. Future research should also explore whether neurodevelopmental difficulties (particularly autism), present differently across ethnicities, and whether screening tools, developed for a predominantly Western White male population, need revalidating across racialised groups.

### Implications for clinical care

Clinicians and partner services must be reflective and mindful of certain structural biases inherent to their institution/practice that might disadvantage young people from certain racialised groups, making these young people less likely to be identified as having a health or social need, and therefore more vulnerable to higher levels of youth justice intervention earlier on than their White counterparts. Conversely, services should also strive to also protect the vulnerability of White youths who have NDD diagnoses. Unconscious bias training should continue to be promoted so that clinicians can consider the young people's experiences (and their family's) through their lens.

Early identification is needed, with professionals seeking to identify factors that might contribute to risk presentations among young people from Black, Dual Heritage and Arab backgrounds, to enable early support. Also, health and justice professionals need to consider vulnerabilities and needs of young people from racialised groups (adopting a trauma-informed approach) when considering how to best proceed with young people from racialised groups before or after arrest. Finally mental health services, including F-CAMHS, can work with local services to increase their awareness of neurodiversity, and support them to challenge stigma about diagnosis in racialised families, which might affect their engagement with mental health services.^18^

## About the authors

**Michelle A. Sandiford** is a principal clinical psychologist with the North West London Forensic Child and Adolescent Mental Health Service, West London NHS Trust, London, UK. **David Moran** is a clinical nurse specialist with the North West London Forensic Child and Adolescent Mental Health Service, West London NHS Trust, London, UK. **Jared G. Smith** is a senior research fellow at the Population Health Research Institute, St George's, University of London, UK. **Heidi Hales** was a consultant child and adolescent forensic psychiatrist in adolescent and forensic psychiatry with the West London Community Forensic Child and Adolescent Mental Health Service Team, West London NHS Trust, London, UK; she is a consultant child and adolescent forensic psychiatrist with the Conwy Child and Adolescent Mental Health Service at Betsi Cadwaladr University Health Board North Wales, Bangor, UK; and is an honorary senior lecturer at the School of Medicine, Cardiff University, UK.

## Data Availability

Data available from the corresponding author, M.A.S., on request due to privacy/ethical restrictions.
